# In vivo functional analysis of *Drosophila* Robo1 immunoglobulin-like domains

**DOI:** 10.1186/s13064-016-0071-0

**Published:** 2016-08-18

**Authors:** Marie C. Reichert, Haley E. Brown, Timothy A. Evans

**Affiliations:** 1 Department of Biological Sciences, University of Arkansas, Fayetteville, AR 72701 USA; 2 Present address: Intramural Research Training Program, National Human Genome Research Institute, Bethesda, MD 20892 USA

**Keywords:** *Drosophila*, Slit, Robo, Axon guidance, Midline crossing, Immunoglobulin-like domain

## Abstract

**Background:**

In animals with bilateral symmetry, midline crossing of axons in the developing central nervous system is regulated by Slit ligands and their neuronal Roundabout (Robo) receptors. Multiple structural domains are present in an evolutionarily conserved arrangement in Robo family proteins, but our understanding of the functional importance of individual domains for midline repulsive signaling is limited.

**Methods:**

We have examined the functional importance of each of the five conserved immunoglobulin-like (Ig) domains within the *Drosophila* Robo1 receptor. We generated a series of Robo1 variants, each lacking one of the five Ig domains (Ig1-5), and tested each for their ability to bind Slit when expressed in cultured *Drosophila* cells. We used a transgenic approach to express each variant in *robo1’s* normal expression pattern in wild-type and *robo1* mutant embryos, and examined the effects of deleting each domain on receptor expression, axonal localization, regulation, and midline repulsive signaling in vivo.

**Results:**

We show that individual deletion of Ig domains 2–5 does not interfere with Robo1’s ability to bind Slit, while deletion of Ig1 strongly disrupts Slit binding. None of the five Ig domains (Ig1-5) are individually required for proper expression of Robo1 in embryonic neurons, for exclusion from commissural axon segments in wild-type embryos, or for downregulation by Commissureless (Comm), a negative regulator of Slit-Robo repulsion in *Drosophila.* Each of the Robo1 Ig deletion variants (with the exception of Robo1∆Ig1) were able to restore midline crossing in *robo1* mutant embryos to nearly the same extent as full-length Robo1, indicating that Ig domains 2–5 are individually dispensable for midline repulsive signaling in vivo.

**Conclusions:**

Our findings indicate that four of the five Ig domains within *Drosophila* Robo1 are dispensable for its role in midline repulsion, despite their strong evolutionary conservation, and highlight a unique requirement for the Slit-binding Ig1 domain in the regulation of midline crossing.

## Background

### Slits and Robos regulate midline crossing in bilaterian animals

The proper establishment of connectivity across the midline of the central nervous system (CNS) is essential for bilateral coordination in a wide variety of animal groups [1]. During embryonic development, CNS axons must choose whether or not to cross the midline in response to attractant and repellant cues produced by midline cells. Axon guidance receptors of the Roundabout (Robo) family regulate midline crossing by signaling midline repulsion in response to their canonical ligand Slit [2]. While the core components of the Slit-Robo pathway (one or more Slits signaling through one or more Robo receptors) are evolutionarily conserved across bilaterian phyla [3–13], the number and identity of pathway components varies, and distinct regulatory mechanisms have appeared in different animal groups [14–16].

### Slit-Robo signaling in *Drosophila*

Robo1 is the primary Slit receptor in *Drosophila*, and normally non-crossing axons ectopically cross the midline in every segment of the embryonic CNS in *robo1* null mutants [3, 17]. Robo1 is broadly expressed in the *Drosophila* embryonic CNS, yet the majority of CNS axons will cross the midline [3, 18]. Two regulatory mechanisms have been identified which prevent premature Slit-Robo1 repulsion in pre-crossing commissural axons in *Drosophila*. The endosomal sorting receptor Commissureless (Comm) prevents newly synthesized Robo1 proteins from reaching the growth cone surface as commissural axons are growing towards and across the midline [14, 19–21], and Robo2 acts non-autonomously to antagonize repulsive signaling by the remaining surface-localized Robo1, facilitating midline crossing [15]. Comm also appears to regulate Robo1 through an additional mechanism that is independent of endosomal sorting, but this role is not well understood [22]. Orthologs of Comm and Robo2 have not been identified outside of insects, and vertebrates have acquired distinct regulatory mechanisms to prevent premature Slit-Robo repulsion in commissural axons [16, 23].

### Conserved structure of Robo receptors and functional modularity of Ig domains

Nearly all Robo family receptors in insects, mammals, nematodes, and planarians share a conserved protein structure, with five immunoglobulin-like (Ig) domains and three fibronectin type III (Fn) repeats making up each receptor’s ectodomain [3, 5, 8, 10, 24–26]. The exceptions to this rule are mammalian Robo4/Magic Roundabout, which lacks Ig3, Ig4, Ig5, and Fn1 [27], and Robo1a/Robo1b from the silkworm *Bombyx mori*, which lack Ig5 and Fn1 [11].

In vitro biochemical interaction and co-crystallization studies have shown that the N-terminal Ig1 domain is the primary Slit-binding region in both insect and mammalian Robo receptors [28–33], and in vivo studies demonstrate the functional importance of Ig1 for midline repulsive activity of both *Drosophila* Robo1 and Robo2 [15, 34]. Functional roles for other extracellular Robo domains in contexts other than Slit-dependent midline repulsion have been described. For example, *Drosophila* Robo2’s Ig2 domain contributes to its role in promoting midline crossing [15, 35], while Robo2’s Ig3 domain has been implicated in regulating longitudinal pathway formation in the *Drosophila* embryonic CNS [35]. In mammals, the divergent Robo3/Rig-1 receptor does not bind Slit [33], but interacts with the novel ligand Nell2 in an Fn-dependent manner to steer commissural axons towards the midline of the embryonic mouse spinal cord [36].

### An in vivo structure/function analysis of all five Robo1 Ig domains

Although it is clear that the various axon guidance activities of Robo family members depend on individual functional domains within the receptor, or combinations thereof, we do not yet have a clear picture of how each domain contributes to individual axon guidance events. Apart from Ig1, which of the other domains in *Drosophila* Robo1 are required for midline repulsion, if any? Are any of the other Robo1 Ig or Fn domains required for receptor expression, protein stability, axonal localization, or Slit binding? Here, we address these questions by individually deleting each of the five Robo1 Ig domains and examining the effects of these deletions on Slit binding as well as in vivo protein expression, localization, and Slit-dependent midline repulsive signaling. We use a previously-established genetic rescue assay [34, 37] to remove endogenous *robo1* function and systematically replace it with *robo1* variants from which individual Ig domain coding sequences have been deleted. We find that Ig domains 2–5 of Robo1 are individually dispensable for Slit binding, receptor expression and axonal localization, regulation by Comm, and midline repulsive signaling activity. Our results indicate that the Slit-binding Ig1 domain is the only immunoglobulin-like domain that is individually required for Robo1’s role in midline repulsion during development of the *Drosophila* embryonic CNS.

## Methods

### Molecular biology

#### Robo1 Ig domain deletions

Individual Robo1 Ig domain deletions were generated via site-directed mutagenesis using Phusion Flash PCR MasterMix (Thermo Scientific), and completely sequenced to ensure no other mutations were introduced. Robo1 deletion variants include the following amino acid residues, relative to Genbank reference sequence AAF46887: Robo1∆Ig1 (L153-T1395); Robo1∆Ig2 (P56-V152/V253-T1395); Robo1∆Ig3 (P56-Q252/P345-T1395); Robo1∆Ig4 (P56-P344/E441-T1395); Robo1∆Ig5 (P56-D440/G535-T1395).

#### pUAST cloning

*Robo1* coding sequences were cloned as BglII fragments into p10UASTattB for S2R+ cell transfection. All *robo1* p10UASTattB constructs include identical heterologous 5′ UTR and signal sequences (derived from the Drosophila *wingless* gene) and an N-terminal 3xHA tag. To make *P{10UAS-Comm}86FB*, the entire *comm* coding sequence (plus 163 bp of the 5’ untranslated region) was cloned as an EcoRI-XbaI fragment into p10UASTattB without heterologous leader sequences or epitope tags.

#### robo1 rescue construct cloning

Construction of the *robo1* genomic rescue construct was described previously [34]. Full-length and variant Robo1 coding sequences were cloned as BglII fragments into the BamHI-digested backbone. Robo1 proteins produced from this construct include the endogenous Robo1 signal peptide, and the 4xHA tag is inserted directly upstream of the first Ig domain (Ig2 in Robo1∆Ig1; Ig1 in all other constructs).

### Genetics

The following *Drosophila* mutant alleles were used: *robo1*^*1*^ (also known as *robo*^*GA285*^). The following *Drosophila* transgenes were used: *P{GAL4-elav.L}3 (elavGAL4)*, *P{10UAS-Comm}86FB*, *P{robo1::HArobo1}* [34], *P{robo1::HArobo1∆Ig1}* [34], *P{robo1::HArobo1∆Ig2}*, *P{robo1::HArobo1∆Ig3}*, *P{robo1::HArobo1∆Ig4}*, *P{robo1::HArobo1∆Ig5}*. Transgenic flies were generated by BestGene Inc (Chino Hills, CA) using ΦC31-directed site-specific integration into attP landing sites at cytological position 86FB (for UAS-Comm) or 28E7 (for *robo1* genomic rescue constructs). *robo1* rescue transgenes were introduced onto a *robo1*^*1*^ chromosome via meiotic recombination, and the presence of the *robo1*^*1*^ mutation was confirmed in all recombinant lines by DNA sequencing. All crosses were carried out at 25 °C.

### Slit binding assay

*Drosophila* S2R+ cells were cultured at 25 °C in Schneider’s media plus 10 % fetal calf serum. To assay Slit binding, cells were plated on poly-L-lysine coated coverslips in six-well plates (Robo-expressing cells) or 75 cm^2^ cell culture flasks (Slit-expressing cells) at a density of 1-2 × 10^6^ cells/ml, and transfected with pRmHA3-GAL4 [38] and HA-tagged p10UAST-Robo or untagged pUAST-Slit plasmids using Effectene transfection reagent (Qiagen). GAL4 expression was induced with 0.5 mM CuSO_4_ for 24 h, then Slit-conditioned media was harvested by adding heparin (2.5 ug/ml) to Slit-transfected cells and incubating at room temperature for 20 min with gentle agitation. Robo-transfected cells were incubated with Slit-conditioned media at room temperature for 20 min, then washed with PBS and fixed for 20 min at 4 °C in 4 % formaldehyde. Cells were permeabilized with PBS + 0.1 % Triton X-100, then stained with antibodies diluted in PBS + 2 mg/ml BSA. Antibodies used were: mouse anti-SlitC (Developmental Studies Hybridoma Bank [DSHB] #c555.6D, 1:50), rabbit anti-HA (Covance #PRB-101C-500, 1:2000), Cy3-conjugated goat anti-mouse (Jackson Immunoresearch #115-165-003, 1:500), and Alexa 488-conjugated goat anti-rabbit (Jackson #111-545-003, 1:500). After antibody staining, coverslips with cells attached were mounted in Aqua-Poly/Mount (Polysciences, Inc.). Confocal stacks were collected using a Leica SP5 confocal microscope and processed by Fiji/ImageJ [39] and Adobe Photoshop software.

### Immunohistochemistry

*Drosophila* embryo collection, fixation and antibody staining were carried out as previously described [40]. The following antibodies were used: FITC-conjugated goat anti-HRP (Jackson #123-095-021, 1:100), mouse anti-Fasciclin II (DSHB #1D4, 1:100), mouse anti-βgal (DSHB #40-1a, 1:150), mouse anti-Robo1 (DSHB #13C9, 1:100), rabbit anti-GFP (Invitrogen #A11122, 1:1000), mouse anti-HA (Covance #MMS-101P-500, 1:1000), Cy3-conjugated goat anti-mouse (Jackson #115-165-003, 1:1000), Alexa 488-conjugated goat anti-rabbit (Jackson #111-545-003, 1:500). Embryos were genotyped using balancer chromosomes carrying *lacZ* markers, or by the presence of epitope-tagged transgenes. Ventral nerve cords from embryos of the desired genotype and developmental stage were dissected and mounted in 70 % glycerol/PBS. Fluorescent confocal stacks were collected using a Leica SP5 confocal microscope and processed by Fiji/ImageJ [39] and Adobe Photoshop software.

## Results

### Robo1 Ig domains 2–5 are individually dispensable for Slit binding in cultured *Drosophila* cells

**Fig. 1:**
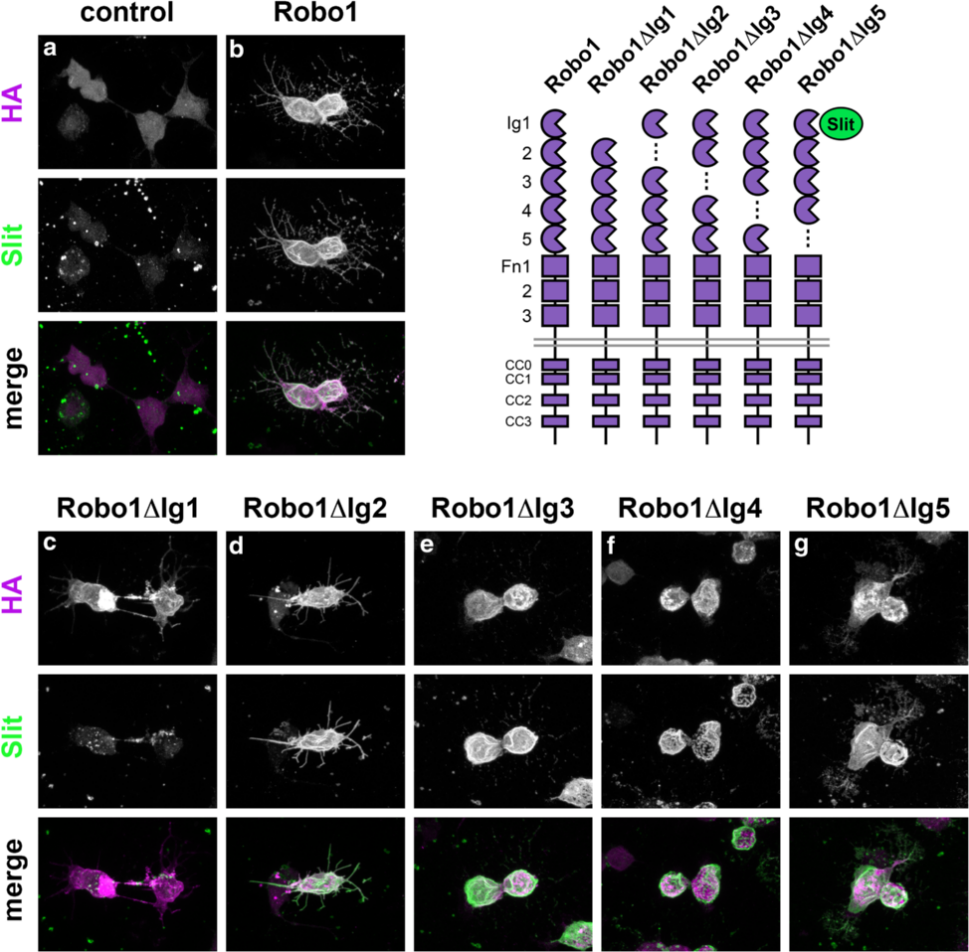
Deletion of individual Robo1 Ig2-5 domains does not interfere with Slit binding in cultured *Drosophila* cells. *Drosophila* S2R+ cells were transfected with the indicated HA-tagged *UAS-Robo1* transgenes, and treated with conditioned media from cells expressing Slit. After Slit treatment, cells were fixed and stained with anti-HA (magenta) to detect expression of Robo1 variants, and anti-Slit (green). Slit binds robustly to cells expressing full-length Robo1 (**b**), but not to mock-transfected cells (**a**) or cells expressing Robo1∆Ig1 (**c**). Cells expressing Robo1∆Ig2 (**d**), Robo1∆Ig3 (**e**), Robo1∆Ig4 (**f**), or Robo1∆Ig5 (**g**) exhibit a similar level of Slit binding to cells expressing full-length Robo1. Schematics of the tested Robo1 variants are shown at *top right*

The Roundabout (Robo) receptor family is an evolutionarily conserved group of transmembrane axon guidance receptors that regulate midline crossing of axons in many bilaterian species. Nearly all Robo receptors share a conserved arrangement of five immunoglobulin-like (Ig) domains and three fibronectin type III (Fn) repeats in their extracellular region. We have recently demonstrated that deletion of the Ig1 domain from *Drosophila* Robo1 prevents it from binding to Slit, and abolishes its ability to prevent midline crossing of axons in vivo [34]. To determine whether Ig domains 2–5 of Robo1 contribute to Slit binding we generated a series of Robo1 variants, each lacking one of the five extracellular Ig domains, and assayed their ability to bind Slit when expressed in cultured *Drosophila* cells. While deletion of the Ig1 domain reduced Slit binding to background levels [34], we found that Robo1∆Ig2, Robo1∆Ig3, Robo1∆Ig4, and Robo1∆Ig5 bound Slit as effectively as full-length Robo1 (Fig. 1). All of the variant receptors were expressed at similar levels and properly localized to the plasma membrane, as assayed by anti-HA staining of transfected cells. Thus, individual deletion of Ig2, Ig3, Ig4, or Ig5 does not affect membrane localization of Robo1 or its ability to interact with Slit.

### Robo1 Ig domains are not individually required for expression and localization in vivo

**Fig. 2:**
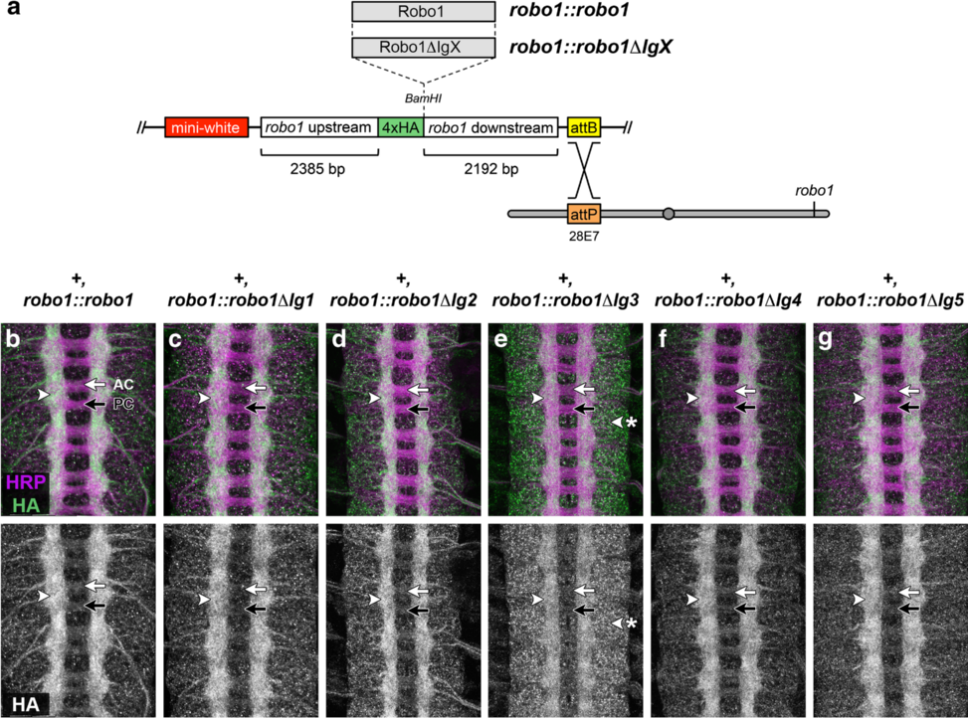
Robo1 Ig2-5 domains are not required for axonal localization and exclusion from commissures in wild-type embryos. **a** Schematic of the *robo1* rescue construct (Brown et al., 2015). HA-tagged Robo1 variants are expressed under the control of regulatory regions from the *robo1* gene. All transgenes are inserted into the same genomic landing site at cytological position 28E7. **b**–**g** Stage 16 embryos stained with anti-HRP (magenta) and anti-HA (green) antibodies. Bottom images show HA channel alone from the same embryos. HA-tagged full-length Robo1 (**b**) and each of the Ig domain deletion variants (**c**–**g**) expressed from the *robo1* rescue transgene in a wild-type background are localized to longitudinal axon pathways (*arrowhead*) and excluded from commissural axon segments in both the anterior commissure (AC, *white arrow*) and posterior commissure (PC, *black arrow*). Robo1^∆Ig3^ expression is elevated within neuronal cell bodies compared to the other transgenes (**e**, *arrowhead* with asterisk)

To compare the expression, localization, and activity of our Robo1 domain deletion variants in vivo, we used a *robo1* genomic rescue construct in which regulatory sequences derived from the endogenous *robo1* locus control expression of HA-tagged cDNAs encoding full-length Robo1 or each of our Robo1 Ig deletion variants (Fig. 2) [34, 37]. All rescue constructs contain identical upstream and downstream regulatory sequences, and all transgenes were inserted into the same genomic location to ensure equivalent expression levels (insertion site 28E7).

We found that all five Robo1 variants were expressed at similar levels to full-length Robo1 and localized to axons in the embryonic ventral nerve cord. Similar to the wild-type Robo1 expression pattern, all five variant Robo1 proteins were detectable across the entire width of the longitudinal connectives, and were strongly downregulated on commissural axon segments (Fig. 2b–g). Indeed the expression patterns of all variants tested here were indistinguishable from the endogenous Robo1 pattern or the HA expression pattern in the full-length Robo1 genomic rescue transgene, with the exception of Robo1∆Ig3. While this variant displayed axonal localization and commissural down-regulation within the neuropile, it also displayed elevated expression in a punctate pattern in the neuronal cell bodies in the cortex (Fig. 2e).

We did not observe any apparent dominant negative effects of expressing any of our Robo1 Ig deletion variants in an otherwise wild-type background, even when present in two copies in homozygous embryos, suggesting that the presence of these variant receptors on the growth cone surface does not alter endogenous Slit-Robo regulation of midline repulsion. Similarly, embryos carrying two copies of any of the rescue transgenes along with two functional copies of endogenous *robo1* did not display any discernible gain-of-function effects (i.e. thinning or loss of commissures indicating increased midline repulsion). This, together with their clearance from commissural axon segments, suggests that the Robo1 Ig deletion variants are subject to the same regulation as endogenous Robo1.

### Regulation of Robo1 Ig deletion variants by Comm

**Fig. 3:**
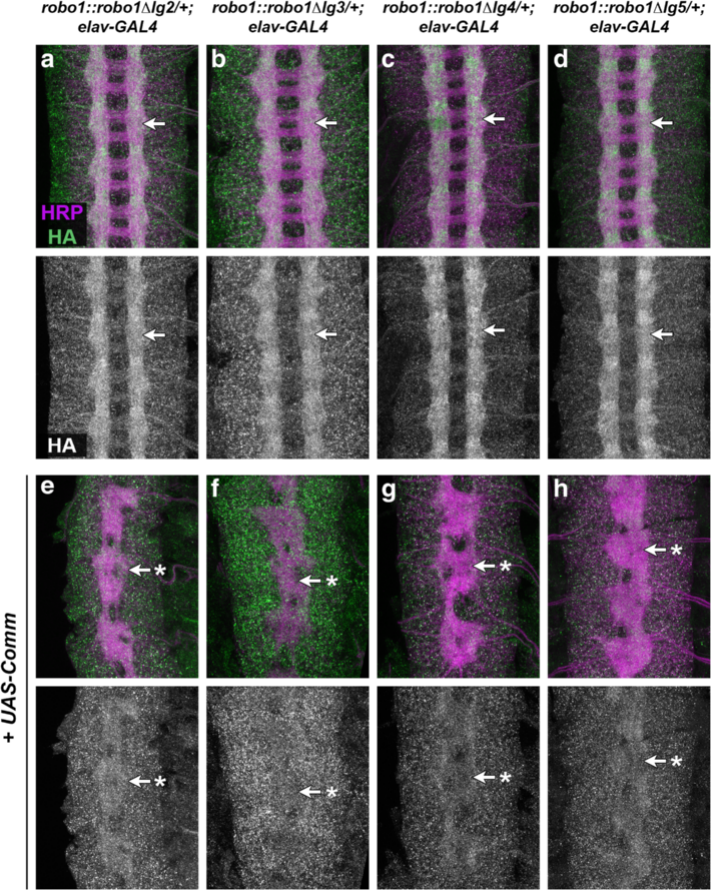
Robo1 Ig domains are not required for regulation by Comm. **a**–**h** Stage 16 embryos stained with anti-HRP (magenta) and anti-HA (green) antibodies. Lower images show HA channel alone from the same embryos. Embryos carrying one copy of the indicated *robo1* transgenes along with *elav-GAL4* display normal expression of the HA-tagged Robo1 variants (**a**-**d**, *arrows*). Embryos carrying one copy of the indicated *robo1* transgenes along with *elav-GAL4* and *UAS-Comm* display strong reduction in axonal HA expression and a *slit-*like midline collapse phenotype reflecting increased midline crossing (**e**-**h**, *arrows* with asterisk). Pairs of sibling embryos shown here (**a** and **e**; **b** and **f**; **c** and **g**; **d** and **h**) were stained in the same tube and imaged using identical confocal settings to allow an accurate comparison of HA levels between embryos

Commissureless (Comm) is an important negative regulator of Slit-Robo1 repulsion in *Drosophila* [14, 19–22]. We have previously reported that the Ig1 domain of Robo1 is not required for regulation of Robo1 by Comm in vivo [34]. To determine whether the other Ig domains of Robo1 are required for Comm-dependent regulation, we examined the effect of Comm misexpression on the expression levels and localization of our Robo1 Ig deletion variants in embryonic neurons. Forced expression of Comm in all embryonic neurons strongly reduces the levels of Robo1 protein on neuronal axons, as Comm is an endosomal sorting receptor that prevents Robo1 protein from reaching the surface of axonal growth cones. We found that for each of our variants, the levels of HA-tagged Robo1 protein on axons were strongly reduced in embryos carrying *elav-GAL4* and *UAS-Comm* compared to embryos carrying *elav-GAL4* alone (Fig. 3). Consistent with down-regulation of both the transgenic and endogenous Robo1 protein, these embryos also displayed a strongly *slit-*like phenotype reflecting high levels of ectopic midline crossing (Fig. 3e–h). These results demonstrate that individually deleting any of the Ig domains from Robo1 does not disrupt Comm-dependent regulation in embryonic neurons.

### Robo1’s Ig2-5 domains are not individually required for midline repulsion in vivo

**Fig. 4:**
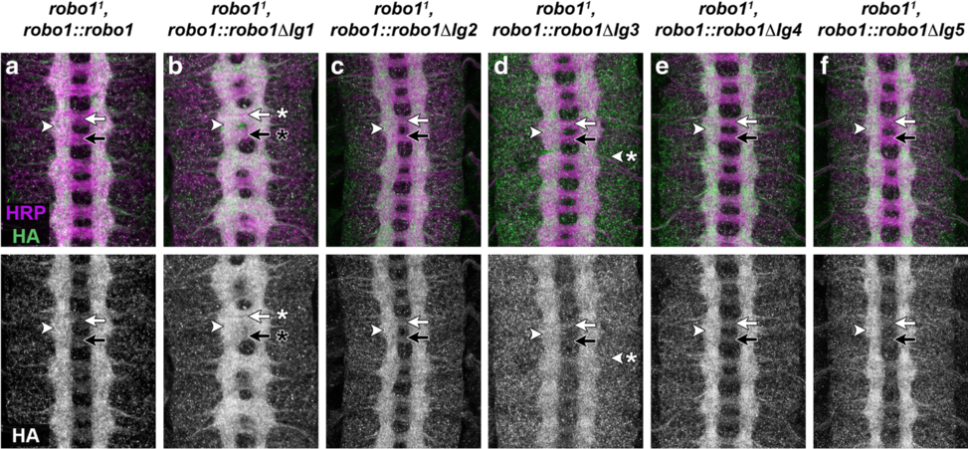
Expression of Robo1 Ig2-5 deletion proteins in *robo1* mutant embryos. **a**–**f** Stage 16 *robo1* mutant embryos carrying indicated *robo1* rescue transgenes, stained with anti-HRP (magenta) and anti-HA (green) antibodies. Lower images show HA channel alone from the same embryos. Expression of full-length Robo1 via the *robo1* rescue transgene in a *robo1* null mutant (**a**) restores the wild-type structure of the axon scaffold, but expression of Robo1∆Ig1 does not (**b**; compare to *robo1* null mutant shown in Fig. 5b). Each of the Ig2-5 deletion variants restore axon scaffold morphology to a similar extent as full-length Robo1 (**c**–**f**). In the absence of endogenous *robo1*, all of the variants are localized to the longitudinal pathways as in wild-type embryos (*arrowheads*) and excluded from the anterior and posterior commissures (*arrows* in **a**, **c**-**f**), with the exception of Robo1∆Ig1 (**b**, *arrows* with asterisks). As in wild-type embryos, Robo1∆Ig3 displays elevated expression levels in neuronal cell bodies compared to the other Robo1 variants (**d**, *arrowhead* with asterisk)

The Slit-binding Ig1 domain of Robo1 is required for its in vivo role in midline repulsion [34]. To test whether Ig domains Ig2-Ig5 are individually required for midline repulsion in vivo, we introduced our *robo1::robo1∆IgX* rescue transgenes into a *robo1* null mutant background and measured their ability to rescue midline repulsion in the absence of endogenous *robo1* activity. Homozygous null *robo1* embryos carrying two copies of our full-length Robo1 rescue transgene exhibited a wild-type axon scaffold, and transgenic HA-tagged Robo1 protein was properly localized to axons and excluded from commissural segments (Fig. 4a), while *robo1* mutant embryos expressing Robo1∆Ig1 phenocopied the *robo1* null phenotype, and transgenic Robo1∆Ig1 protein was detectable on axons as they crossed the midline (Fig. 4b), as previously described [34]. We found that expression of any of our Ig2-5 deletion transgenes in *robo1* null mutants was able to restore the wild-type appearance of the axon scaffold, as measured by anti-HRP staining (Fig. 4c–f). Further, each of the transgenic Robo1 proteins was properly expressed and excluded from commissures in this background, indicating that endogenous *robo1* is not required for proper expression, commissural clearance, or midline repulsive signaling of Robo1∆Ig2, Robo1∆Ig3, Robo1∆Ig4, or Robo1∆Ig5 (Fig. 4c–f). As in a wild-type background, we detected elevated levels of Robo1∆Ig3 in neuronal cell bodies in addition to its axonal expression (Fig. 4d; compare to Fig. 2e).

**Fig. 5:**
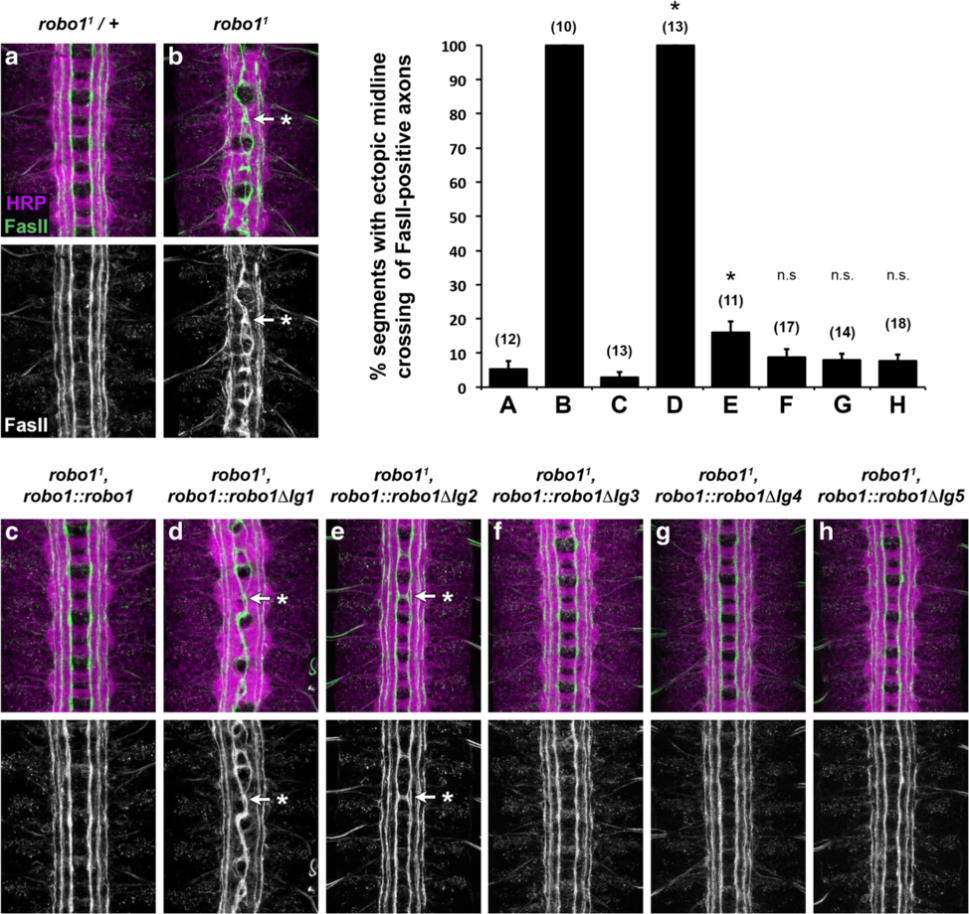
Robo1 Ig2-5 domains are dispensable for midline repulsion in vivo. **a**–**h** Stage 16 embryos stained with anti-HRP (magenta) and anti-FasII (green) antibodies. Lower images show FasII channel alone from the same embryos. FasII-positive axons cross the midline inappropriately in every segment in *robo1* null mutants (**b**, *arrow* with asterisk). This phenotype is completely rescued by a *robo1* genomic rescue transgene expressing full-length Robo1 protein (**c**) but is not rescued by an equivalent rescue transgene expressing Robo1∆Ig1 (**d**). Rescue transgenes expressing each of the four additional Ig deletion variants rescue midline crossing as well as, or nearly as well as, full-length Robo1 (**e**–**h**). When ectopic crossing is observed in these rescue backgrounds, it is less severe than in *robo1* mutants (**e**, *arrow* with asterisk). Bar graph shows quantification of ectopic midline crossing in the genotypes shown in (**a**–**h**). Error bars indicate standard error. The extent of rescue for each Ig deletion variant (**d**–**h**) was compared to *robo1* ^*1*^, *robo1::robo1* embryos (**c**) by Student’s *t*-test, with a Bonferroni correction for multiple comparisons (*p < 0.01 compared to *robo1* ^*1*^, *robo1::robo1*)

To more closely examine the ability of our rescue transgenes to restore midline repulsion in the absence of endogenous *robo1*, we quantified ectopic midline crossing of FasII-positive longitudinal axons in each of our *robo1* rescue backgrounds. In wild-type embryos or *robo1* null mutants rescued with a full-length Robo1 transgene, FasII-positive axons rarely crossed the midline (Fig. 5a, c), but they crossed the midline in 100 % of segments in *robo1* mutants (Fig. 5b). As we have previously reported [34], Robo1∆Ig1 was completely unable to rescue midline repulsion in *robo1* mutant embryos, reflecting the critical role of Robo1 Ig1 in midline repulsion (Fig. 5d). In contrast, we could restore midline repulsion to near-wild-type levels by similarly expressing Robo1∆Ig2, Robo1∆Ig3, Robo1∆Ig4, or Robo1∆Ig5 (Fig. 5e–h). In segments where ectopic crossing was observed in these rescue backgrounds, it was typically less severe than in *robo1* mutants (Fig. 5e, arrow with asterisk).

## Discussion

In this paper, we have examined the functional importance of each of the five immunoglobulin-like (Ig) domains of the *Drosophila* Robo1 axon guidance receptor. We deleted each Ig domain individually and examined the effects on Robo1’s ability to bind its ligand Slit, on expression and localization of Robo1 in the embryonic CNS, and on Robo1’s ability to regulate midline repulsion in vivo. Our results suggest that Ig1 is the only immunoglobulin-like domain in *Drosophila* Robo1 that is indispensable for its midline repulsive activity. Deleting any of the other four Ig domains individually does not alter the structure or confirmation of Robo1 in a way that interferes with Slit binding in vitro or repulsive signaling in vivo. This is consistent with recent evidence that deleting Ig2 from Robo2 does not interfere with its ability to bind Slit or signal midline repulsion [15], and supports a modular view of Robo1 ectodomains wherein individual Ig domains can function independently to promote distinct molecular events (e.g. ligand binding) and cellular outcomes (e.g. axon repulsion) [35].

### Robo1 Ig domains are not individually required for protein stability or axonal localization

Deleting any of the five Ig domains did not significantly disrupt the expression or axonal localization of Robo1 in embryonic neurons, suggesting no large effects on protein stability or folding (Fig. 2b–g). HA expression in wild-type embryos carrying each of the Ig deletion variants was largely indistinguishable from full-length HA-tagged Robo1, or endogenous Robo1 protein expression, with the exception of Robo1∆Ig3. This variant displayed axonal expression levels that were roughly equivalent to full-length Robo1 and the other Ig deletion variants, but was also detectable at increased levels within neuronal cell bodies (Fig. 2e). Notably, Robo1∆Ig3 did not appear to localize to the cell body plasma membrane, but remained within intracellular puncta, presumably vesicles within the protein synthesis and transport pathway. The levels of axonal Robo1∆Ig3 appear to be sufficient for normal signaling activity, as this variant rescued midline repulsion equally as well as the other Ig deletion variants (Fig. 5f).

All five Robo1 Ig deletion variants were cleared from commissures when expressed in otherwise wild-type embryos, and we did not observe any obvious gain of function or dominant negative effects caused by their expression, as the axon scaffold appeared normal in embryos carrying two copies of any of the five rescue transgenes when visualized with anti-HRP antibody staining (Fig. 2c*–*g).

### Does Ig2 contribute to Slit binding or midline repulsion?

Notably, Robo1∆Ig2 was the only deletion variant (other than Robo1∆Ig1) whose ability to rescue *robo1* mutants was significantly different than full-length Robo1, suggesting that Ig2 may contribute to Slit binding and/or repulsive signaling, though to a lesser extent than Ig1 (Fig. 5e). Previous in vitro experiments suggested that Ig2 is required for Slit binding by human Robo1 [29], while other experiments suggested that Ig2 does not contribute to Slit binding [32, 41]. While we did not detect any qualitative differences in Slit binding between full-length Robo1 and Robo1∆Ig2 in our cell culture-based experiments (Fig. 1b, d), perhaps a quantitative difference in Slit affinity might be detected using more sensitive assays [30–32, 35]. Even if Ig2 does not directly contribute to Slit binding, it may help to stabilize or enhance interactions with Slit or heparin, which forms a ternary complex with Slit and Robo and contributes to Slit-Robo signaling [42–45]. In previous studies, site-specific mutations of evolutionarily conserved residues in Ig2 of *Drosophila* Robo1 had minor effects on binding of Slit or heparin to Robo1 in vitro [32]; perhaps this could account for the slight but significant reduction in midline repulsive activity of our Robo1∆Ig2 variant.

### Signaling mechanisms of Robo family receptors

Robo family receptors are transmembrane proteins which lack intracellular catalytic domains, and the mechanisms through which they signal axon repulsion are not well characterized. Although it is known that cytoplasmic effector proteins are recruited to the Robo1 cytodomain upon Slit binding [46, 47] and that proteolytic processing and endocytosis of Robo1 are necessary for repulsive signaling [48, 49], it is unknown whether ligand binding induces a change in multimerization state, or some other type of conformational change in order to trigger downstream signaling events. It is also unknown how (or even whether) the extracellular domains apart from Ig1 contribute to the signaling mechanism(s). Perhaps Ig domains 2–5, though not individually required for midline repulsion, serve as “spacers” to position the Slit-binding Ig1 domain at a particular distance from the cell membrane or to facilitate a particular conformational change within the ectodomain upon Slit binding. If this is the case, the requirement must not be a strict one because we can delete any single Ig domain in between Ig1 and the transmembrane region without severely compromising Robo1’s ability to signal. In this context, it is worthwhile to note that Ig1 and Ig2 are the most strongly conserved in terms of sequence identity, with 58 % and 48 % identity between *Drosophila* Robo1 and human Robo1 for Ig1 and Ig2, respectively [3]. The sequences of Ig 3–5 are less highly conserved (35 % identity for each of the three domains between *Drosophila* Robo1 and human Robo1), perhaps indicating that their three-dimensional structure or arrangement might be more important than their amino acid sequence. It will be interesting to determine how many, or what combination of Ig domains can be removed without disrupting midline repulsive signaling. In vitro structural studies will likely be required (for example, a structural comparison of the entire Robo1 ectodomain in liganded and unliganded states) to fully understand how each domain contributes to Slit-dependent signaling.

### Evolutionary conservation of Robo receptor Ig domains

Nearly all Robo family receptors share *Drosophila* Robo1’s 5 Ig + 3 Fn ectodomain structure. The Ig1 domain of *Drosophila* Robo1 is absolutely required for Slit binding and midline repulsive activity in vivo [34]; Ig1 domains in other Robo receptors appear to have equally important roles in Slit binding [15, 31, 32]. In contrast, Ig domains 2–5 appear to be individually dispensable for Slit binding and midline repulsive activity, at least in the case of *Drosophila* Robo1 (this study). If the other four Ig domains are dispensable for midline repulsion, why is their number and arrangement so strongly evolutionarily conserved? One possibility is that they are required for signaling by Robo1 in contexts other than midline repulsion of axons, for example embryonic muscle migration [50], migration of embryonic chordotonal sensory neurons [51], or guidance and targeting of dendrites [52–56], or for midline repulsion of axons in other developmental stages or tissues not examined here, for example gustatory receptor neurons in the adult [57]. Another possibility is that one or more of these domains are required for regulation by Robo2, which inhibits Slit-Robo1 repulsion to promote midline crossing [15]. Robo2-dependent defects in midline crossing are evident only when attractive Netrin-Frazzled signaling is also compromised in *robo2* mutants [15, 37], so we would not necessarily expect to observe a decrease in midline crossing if any of our Robo1 Ig deletion variants were insensitive to Robo2. Future studies will examine the effects of misexpressing Robo2 or removing *fra* function in each of the rescue backgrounds described here, which may provide further insight into how Robo2 inhibits Robo1 to promote midline crossing of commissural axons.

## Conclusions

We have described here a systematic functional analysis of all five immunoglobulin-like domains in the *Drosophila* Robo1 axon guidance receptor. This work is the first in vivo study of the functional importance of Robo1 Ig domains other than the Slit-binding Ig1 domain. We have shown that Ig domains 2–5 are not required for Slit binding, and that despite their strong evolutionary conservation, Ig 2–5 are individually dispensable for *Drosophila* Robo1’s in vivo role in regulating midline repulsion in the embryonic CNS. These observations indicate that Ig1 is the only Ig domain in *Drosophila* Robo1 that is uniquely required for midline repulsion, and suggest that the mechanism by which Robo1 signals axon repulsion is not strictly dependent on the evolutionarily conserved 5 Ig + 3 Fn ectodomain structure that is characteristic of Robo family receptors.

## Abbreviations

CNS: Central nervous system
Comm: Commissureless
Fn: Fibronectin type III repeat
Ig: Immunoglobulin-like domain
Robo: Roundabout
